# Supplementary material for: Movement quantity and quality: How do they relate to pain and disability in dancers?
Source: PLoS One. 2022 May 19;17(5):e0268444. doi: 10.1371/journal.pone.0268444 (PMC9119517; doi:10.1371/journal.pone.0268444)
Supplement: Attachment — Submitted filename: Response to the editor.docx [file pone.0268444.s001.docx]

**Response to the editor: 11 March 2022**

**Editors comment:** 1. We note you state the data for this study are available upon request. Please note, in line with our goal of ensuring long-term data availability to all interested researchers, PLOS’ Data Policy states that authors cannot be the sole named individuals responsible for ensuring data access (<http://journals.plos.org/plosone/s/data-availability#loc-acceptable-data-sharing-methods>).

Before we proceed with your manuscript, please address the following prompts:

a) If there are ethical or legal restrictions on sharing a de-identified data set, please explain them in detail (e.g., data contain potentially identifying or sensitive patient information, data are owned by a third-party organization, etc.) and who has imposed them (e.g., a Research Ethics Committee or Institutional Review Board, etc.). Please also provide non-author contact information for a data access committee, ethics committee, or other institutional body to which data requests may be sent.

**Authors Response:** We thank the editor for the opportunity to update this. All data is available for verification or other research following approval that such use complies with the consent provided by participants. Researchers wishing to access the data can seek approval from the Curtin University Human Ethics Committee quoting project approval number HREC2017-0726. The contact for the Curtin University Human Ethics Committee is +61 8 9266 9223 or ethics@curtin.edu.au. This has been included in the revised cover letter
